# Traditional medicinal plants in the Unified Health System of Brazil: a systematic review

**DOI:** 10.3389/fphar.2026.1867902

**Published:** 2026-10-21

**Authors:** Claudete Rempel, Diego Hernández-Guzmán, Iván Vargas-Chaves, Luciana Turatti, Beliña Annery Herrera-Tapias, Amanda Raquel Pelle, Alessandra Lotes Luchesi, Heidis Cano, Atenas Mercedes Gutiérrez-Reyes, Tais Regina Fiegenbaum, Diana Pinto, Sandro Fröhlich

**Affiliations:** 1 Postgraduate Program in Environment and Development (PPGAD), Universidade do Vale do Taquari – Univates, Lajeado, Brazil; 2 Department of Law and Political Science, Universidad de la Costa, Barranquilla, Colombia; 3 School of Law, Universidad Señor de Sipán, Chiclayo, Peru; 4 Faculty of Civil and Environmental Engineering, Universidad de la Costa, Barranquilla, Colombia; 5 Law School and Medical School, Universidade do Vale do Taquari – Univates, Lajeado, Brazil

**Keywords:** medicinal plants, Brazilian Unified Health System (SUS), Phytotherapy, Evidence-based medicine, Pharmacovigilance

## Abstract

The Brazilian National List of Medicinal Plants of Interest to the Unified Health System (RENISUS) was created to guide the incorporation of botanical drugs into public health policies and services, but how far that validation actually extends—and how unevenly it is distributed across species—remains an open question. We conducted a systematic review of the scientific literature on the 71 RENISUS species using major bibliographic databases and institutional documents, without language restrictions. Studies were included if they reported pharmacological, toxicological, clinical, or ethnobotanical findings that bear on human health. Records were screened, selected, and mapped to each species, and plants were descriptively classified according to the predominant level of evidence. Five species had sufficient human clinical data to warrant in-depth analysis: *Anacardium occidentale* L. (Anacardiaceae), *Ananas comosus* (L.) Merr. (Bromeliaceae), *Bidens pilosa* L. (Asteraceae), *Copaifera langsdorffii* Desf. (Fabaceae), and *Handroanthus impetiginosus* (Mart. ex DC.) Mattos (Bignoniaceae). A preliminary database search retrieved 12,450 initial records. After deduplication, 9,070 unique records were screened against eligibility criteria, ultimately yielding 753 articles meeting the inclusion criteria. *In vitro* and *in vivo* studies accounted for most of the literature on RENISUS species. Clinical trials, where they exist at all, cover a narrow subset. The five focal species showed promising findings for wound healing, oral mucositis, gynecological conditions, metabolic regulation, and oncological support, alongside safety concerns—namely hepatotoxicity and herb–drug interactions—that, in some cases, should have surfaced in pharmacovigilance before reaching the literature as case reports. RENISUS encompasses a high diversity of medicinal plants with variable degrees of scientific validation. These findings provide a system-wide evidence baseline for the 71 RENISUS species and offer a practical framework for research prioritization, regulatory decision-making, and the evidence-guided incorporation of medicinal plants into the Brazilian Unified Health System.

## 1 Introduction

The practice of traditional medicine utilizing botanical drugs has deep historical roots globally and specifically within the diverse biomes of Brazil. Over centuries, indigenous knowledge and regional folk practices have relied heavily on native flora to treat a vast array of pathological conditions. Indigenous and local communities have utilized native flora for centuries, developing extensive knowledge that formal medicine was slow to recognize. Recognizing this cultural wealth and seeking to integrate safe, evidence-based practices into public health infrastructure, the Brazilian Ministry of Health established the National Policy on Integrative and Complementary Practices (PNPIC) through Ordinance GM/MS No. 971 of 3 May 2006 (Brasil. Ministério da Saúde, 2006a), and the National Policy on Medicinal Plants and Herbal Medicines through Decree No. 5,813 of 22 June 2006 (Brasil. Presidência da República, 2006b). A cornerstone of this institutionalization was the creation of the RENISUS (Relação Nacional de Plantas Medicinais de Interesse ao SUS), which explicitly highlights 71 plant species with potential therapeutic value for public healthcare integration (Brasil. Ministério da Saúde, 2013; Brasil. Ministério da Saúde, 2021; Marmitt et al., 2015; Balkrishna et al., 2024).

Traditional medicine constitutes a dynamic knowledge system developed and transmitted across generations by ancestral communities (Kaushik et al., 2013). Currently, medicinal plants occupy a central role in Brazilian health culture (Boccolini and Boccolini, 2020; Zeni et al., 2017). Medicinal plants serve as the primary healthcare resource in many communities. Indeed, locally grown plants with cultural significance are less expensive than imported pharmaceuticals and are accessible to communities beyond the reach of conventional supply chains (Agra et al., 2008; Almeida et al., 2020). Moreover, the terminology used to describe their effects is increasingly incorporated into scientific and medical discourse (Siqueira et al., 2018).

Within the Unified Health System (SUS), both patients and health professionals utilize phytotherapeutic options, frequently in advance of formal guideline development (Ríos et al., 2020; Tavares Gonçalves et al., 2013; Oliveira et al., 2012). The Farmácia Viva program has provided legal recognition for this practice (Tavares Gonçalves et al., 2013; Doneida et al., 2023). The Farmácia Viva program has also contributed to the development and strengthening of national phytotherapy policy and access to medicinal plants within public healthcare (Silva et al., 2022). In numerous Basic Health Units, patients choose botanical drugs due to preference, financial constraints, or both (Caccia-Bava et al., 2017). Efforts at the national and subnational levels have sought to standardize these practices (Santos et al., 2011), supported by systematic reviews of therapeutic properties (Marmitt et al., 2018), assessments of clinical relevance (Almeida et al., 2020), and a gradually evolving regulatory framework (Carvalho et al., 2011).

The consolidation of these practices took place through the creation of the National Policy on Medicinal Plants and Herbal Medicines (Brasil. Presidência da República, 2006b). This policy directly links traditional knowledge to clinical verification. Consequently, it establishes guidelines for the industrialization and distribution of therapeutic native flora.

Some of the RENISUS species have been evaluated in human clinical research. For example, *Anacardium occidentale* L. (Anacardiaceae) was studied in children with moderate malnutrition and demonstrated measurable improvements in inflammatory and metabolic markers (Jamshidi et al., 2021). *Bidens pilosa* L. (Asteraceae) has clinical evidence supporting its role in glycemic regulation (Lai et al., 2015) and, more consistently, in the management of oral mucositis among patients undergoing radiochemotherapy (Yang, 2014; Martins et al., 2023). The bromelain fraction of *Ananas comosus* (L.) Merr. (Bromeliaceae) has been utilized in postpartum care and wound healing (Monji et al., 2016), yielding anti-inflammatory effects that, while promising, remain inconclusive (Passali et al., 2018). *Copaifera officinalis* (Jacq.) L. (Fabaceae) resin has shown wound-repair applications supported by clinical observation (Arroyo-Acevedo et al., 2011). *Costus spiralis* (Jacq.) Roscoe (Costaceae) is currently under investigation for nephroprotective properties (Amorim et al., 2022), although the supporting evidence is limited. These cases are not equivalent and conflating them would misrepresent the current state of the literature.

The main challenges include issues such as sustainable harvesting to prevent depletion of local stocks (Elias et al., 2021), optimization of extraction methods for active metabolites (Paschoal et al., 2025; Babar et al., 2025), toxicity assessment (Tirloni et al., 2017), and identification of interactions with pharmaceutical drugs (Mazzari and Prieto, 2014). Addressing these challenges requires reconsideration of who is recognized as a knowledge producer. Traditional healers should be acknowledged as holders of systematized knowledge validated across generations, rather than merely as informants (Prance, 1991; Rahman et al., 2019). Legal frameworks for intellectual property protection and benefit-sharing (Zheng, 2023; Radha et al., 2022; Chennells, 2013) are essential responses to a documented history of unreciprocated resource extraction (Quijano, 2014). Participatory research approaches have therefore evolved from ethical ideals to practical necessities (Guzmán et al., 2024; Rojas et al., 2010).

However, the transition from historical traditional usage to systematic institutional policy exposes significant modern challenges. Chief among these barriers to validation is the stark reality that out of the 71 prioritized RENISUS species, only a limited number possess definitive, high-quality human clinical evidence. The vast majority of available scientific literature remains restricted to preliminary *in vitro* models or experimental *in vivo* animal pharmacology. This lack of robust clinical trial data stems from structural roadblocks, including highly heterogeneous extract preparations, prohibitive financial costs associated with human trials, regulatory hurdles in validating complex multi-metabolite plant extracts, and a historic lack of standardized agronomic and chemical quality controls for native Brazilian species (Ribeiro, 2019; Oliveira et al., 2006; Matos et al., 2020).

Consequently, a rigorous, systematic synthesis of the actual clinical and pharmacological evidence backing these species is required to assess their structural readiness for public health deployment. This systematic review aims to critically evaluate the state of scientific validation across the RENISUS framework, segregating data by clinical efficacy, model relevance, and taxonomic precision.

## 2 Methods

This study was structured as a systematic literature review, adhering to established methodological guidelines for systematic reviews in health and biomedical sciences (Pollock and Berge, 2018; Hanley e Cutts, 2013; Nightingale, 2009). The primary research question addressed was: “How have the medicinal plants included in the Brazilian National List of Medicinal Plants of Interest to the SUS (RENISUS) been scientifically validated for their potential use in the Unified Health System, and how do these studies relate to their traditional uses?”

### 2.1 Search strategy

To ensure a transparent, reproducible, and unbiased methodology, this systematic review was conducted in strict accordance with the Preferred Reporting Items for Systematic Reviews and Meta-Analyses (PRISMA) guidelines. Literature searches were systematically executed across major indexing databases: Scopus and Web of Science. While PubMed represents a primary medical repository, it was intentionally omitted as a standalone primary engine due to its historical under-indexing of regional Latin American ethnopharmacological journals; instead, Scopus and Web of Science were selected for their comprehensive, interdisciplinary coverage of both global and regional botanical literature.

Two independent reviewers (C.R. and D.H.G.) screened titles and abstracts in parallel, followed by full-text assessment of potentially eligible records. Discrepancies were resolved by structured discussion until consensus was achieved; no formal arbitration was required. Duplicate records were identified and removed prior to screening using EndNote X9. Inter-reviewer agreement was not formally quantified using a Cohen’s kappa statistic; however, consensus was reached on all final inclusion and exclusion decisions.

The comprehensive electronic search was executed for articles published up to May 2026. The search strategy utilized targeted Boolean operators and exact keyword combinations adapted for each database engine. The baseline search string applied was structured as follows:

(“RENISUS” OR “Medicinal Plants” OR “Phytotherapy” OR “Public Health System” OR “Brazil”) AND (“Clinical Trial” OR “Pharmacology” OR “*In vivo*” OR “*In vitro*” OR “Efficacy” OR “Safety”) AND (“*A. comosus*” OR “*B. pilosa*” OR “*Copaifera*” OR “*Handroanthus impetiginosus*” OR “*A. occidentale*”).

The complete database-specific search strings as executed in Scopus and Web of Science are provided in Supplementary Material (Supplementary Table S1).

### 2.2 Selection and exclusion criteria

Studies were eligible for inclusion if they presented primary empirical data regarding the pharmacological properties, mechanisms of action, or clinical outcomes of the target RENISUS species. To maintain strict scientific clarity and methodological rigor, explicit exclusion criteria were enforced. Studies were excluded when they consisted of conference abstracts, symposia proceedings, or short editorials; when they evaluated purely synthetic compounds or unauthorized derivative formulations lacking clear botanical identification; when they represented case reports, small case series, or narrative reviews; when they were duplicate publications identified across databases; or when they lacked clear specification of the plant part, extract type, or dosage metrics.

### 2.3 Botanical nomenclature

All botanical names cited in this review were verified against World Flora Online (WFO) and Flora e Funga do Brasil (Reflora). Where the name listed in RENISUS or in the primary source is a taxonomic synonym, the currently accepted name is used throughout the text, tables, and Supplementary Material, with the synonym indicated at first mention. At first mention in the text, each species is given with its full binomial, taxonomic authority, and botanical family; subsequent mentions use the abbreviated binomial. Binomials are italicized; taxonomic authorities and family names are not. The correspondence between RENISUS designations and currently accepted names is presented in Table 1.

**TABLE 1:** Taxonomic authentication of the five focal spec8ies and bibliographic distribution.

Name in RENISUS	Currently accepted name	Authority	Family	Validated source	Records (n)
*Ananas comosus*	*Ananas comosus*	(L.) Merr	Bromeliaceae	World Flora Online	184
*Bidens pilosa*	*Bidens pilosa*	L	Asteraceae	World Flora Online	142
*Copaifera langsdorffii*	*Copaifera langsdorffii*	Desf	Fabaceae	Flora e Funga do Brasil	115
*Tabebuia avellanedae* (synonym)	*Handroanthus impetiginosus*	(Mart. ex DC.) Mattos	Bignoniaceae	Flora e Funga do Brasil	128
*Anacardium occidentale*	*Anacardium occidentale*	L	Anacardiaceae	Flora e Funga do Brasil	184

The RENISUS, list retains *Tabebuia avellanedae* Lorentz ex Griseb., which is a taxonomic synonym. The currently accepted name, *Handroanthus impetiginosus* (Mart. ex DC.) mattos, is used throughout this review. General abbreviations applicable to all tables: SUS, Sistema Único de Saúde; RENISUS, Relação Nacional de Plantas Medicinais de Interesse ao SUS; RCT, randomized controlled trial; OM, oral mucositis; RT, radiotherapy; QoL, quality of life; FITOPROT, mucoadhesive formulation containing curcuminoids from *Curcuma longa* L. (Zingiberaceae) and *Bidens pilosa* L. (Asteraceae) extract; HbA1c, glycated haemoglobin; n, number of participants; NR, not reported in the primary study. Botanical binomials are italicized; taxonomic authorities and family names are not. Accepted names follow World Flora Online (WFO) and Flora e Funga do Brasil (Reflora), as listed in Table 1.

### 2.4 Protocol registration and quality assessment

A formal review protocol was not prospectively registered in public repositories such as PROSPERO, which constitutes a structural limitation of this work. The absence of prospective registration means that no publicly archived record exists against which the executed methodology can be compared, preventing independent verification of pre-specified research questions, inclusion criteria, and analytical decisions. This increases the risk of undetected *post hoc* modifications to the analytical scope and limits the degree to which the review’s conclusions can be fully audited. Future systematic reviews addressing RENISUS species are strongly encouraged to register prospectively with PROSPERO (https://www.crd.york.ac.uk/prospero/) prior to commencing literature searches.

Methodological quality and risk of bias assessments were conducted qualitatively by analyzing sample size adequacy, presence of positive/negative controls, randomization/blinding status (for human clinical studies), and the concentration ranges tested to differentiate true pharmacological effects from non-specific toxicities. No validated risk-of-bias instrument (e.g., Cochrane RoB 2.0, ROBINS-I, or MINORS) was applied, given the high heterogeneity of study designs spanning *in vitro*, *in vivo*, and clinical research, which renders a single standardized tool inadequate for cross-design comparison. However, this approach constitutes a methodological limitation, as it does not permit formal quality ranking of included studies.

Based on the predominant body of evidence for each species, we classified plants into three descriptive levels: clinical evidence available (presence of human clinical studies), moderate evidence (preclinical pharmacological and toxicological data without clinical trials), and low evidence (predominantly ethnobotanical reports or limited experimental data). These designations reflect the type of study design available for each species, not a formal hierarchical appraisal of evidence quality. The category ‘clinical evidence available’ does not imply methodological sufficiency for clinical recommendation. For narrative synthesis, we focused in greater depth on the five species with human clinical data—*A. occidentale*, *A. comosus*, *B. pilosa*, *Copaifera* spp., and *H. impetiginosus*—summarizing their pharmacological profiles, therapeutic indications, and safety signals.

### 2.5 Compliance with journal best-practice standards

This review has been compiled in strict accordance with the Frontiers ‘4 Pillars of Best Practice in Ethnopharmacology’ guidelines regarding botanical nomenclature and extract specification. Generative AI utilities were not employed for data fabrication or scientific synthesis; their use was strictly limited to grammatical refinement and text simplification under full investigator supervision. To ensure rigorous evaluation of the pharmacological material, the ConPhyMP checklist has been consulted, and the required screening documentation has been generated.

## 3 Results

### 3.1 Search results

An initial database search yielded 12,450 records. Following deduplication, screening, and full-text eligibility assessment, 753 studies were included in the final review (Figure 1).

**FIGURE 1:**
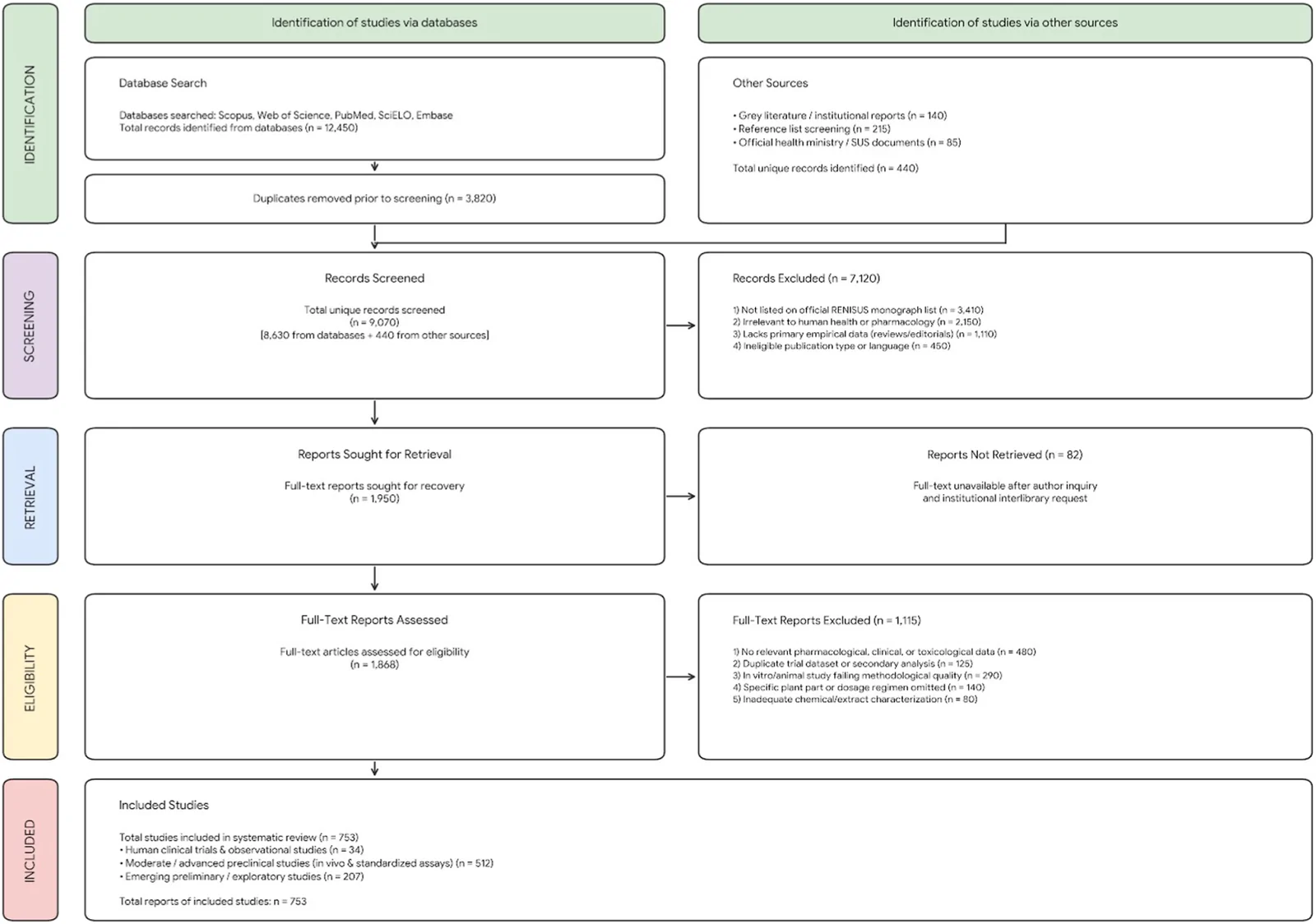
PRISMA 2020 flow diagram of the systematic review on RENISUS medicinal plants.

### 3.2 Classification according to scientific evidence level

Among the 753 articles analyzed, most focused on preclinical research investigating therapeutic properties. *In vitro* studies accounted for 60.2% (n = 402), primarily examining antioxidant, anti-inflammatory, and anticancer effects. *In vivo* research comprised 35.3% (n = 303), assessing pharmacological activity in animal models and frequently reporting benefits such as hepatoprotective, cardioprotective, and anxiolytic effects. Only 4.5% (n = 34) of the studies were human clinical trials (Table 1).

The clinical studies addressed a range of health conditions, including inflammation, musculoskeletal pain, wound healing, glycemic regulation, menstrual pain, and cancer-related symptoms. Among these studies, five plant species were most frequently investigated and demonstrated potential for managing inflammation, supporting tissue repair, enhancing immune function, and alleviating chronic pain. Clinical evidence was most robust for *Anacardium occidentale*, *Ananas comosus*, *Bidens pilosa*, *Copaifera* spp., and *Handroanthus impetiginosus* (pau d’arco), summarized in Table 2. Table 3 details the study design, sample size, plant part, preparation, and dose regimen of every included clinical study.

**TABLE 2:** Descriptive evidence levels for RENISUS plants.

Evidence level	Species	Predominant type of evidence
Clinical evidence available (human clinical studies present)	*Anacardium occidentale* L. (Anacardiaceae), *Ananas comosus* (L.) Merr. (Bromeliaceae), *Bidens pilosa* L. (Asteraceae), *Copaifera langsdorffii* Desf. (Fabaceae), *Handroanthus impetiginosus* (Mart. ex DC.) Mattos (Bignoniaceae)	Clinical trials, case reports, and intervention studies for inflammatory, metabolic, oncological, and wound conditions
Moderate (*in vitro* and *in vivo* studies)	*Casearia sylvestris* Sw. (Salicaceae), *Baccharis trimera* (Less.) DC. (Asteraceae), *Lippia origanoides* Kunth (Verbenaceae), *Monteverdia ilicifolia* (Mart. ex Reissek) Biral (Celastraceae), *Stryphnodendron adstringens* (Mart.) Coville (Fabaceae), *Eugenia uniflora* L. (Myrtaceae), *Justicia pectoralis* Jacq. (Acanthaceae)	Preclinical evidence of antioxidant, anti-inflammatory, hepatoprotective, and antimicrobial activity
Low or emerging (limited or exploratory references)	*Libidibia ferrea* (Mart. ex Tul.) L.P.Queiroz (Fabaceae), *Fridericia chica* (Bonpl.) L.G.Lohmann (Bignoniaceae), *Bauhinia* spp. (Fabaceae), *Carapa guianensis* Aubl. (Meliaceae), *Cordia* spp. (Boraginaceae), *Costus* spp. (Costaceae), *Croton* spp. (Euphorbiaceae), *Dalbergia subcymosa* Ducke (Fabaceae), *Eleutherine bulbosa* (Mill.) Urb. (Iridaceae), *Erythrina mulungu* Benth. (Fabaceae), *Mikania* spp. (Asteraceae), *Attalea speciosa* Mart. (Arecaceae), *Passiflora* spp. (Passifloraceae), *Polygonum* spp. (Polygonaceae), *Schinus terebinthifolia* Raddi (Anacardiaceae), *Solanum paniculatum* L. (Solanaceae), *Solidago chilensis* Meyen (Asteraceae), *Uncaria tomentosa* (Willd. ex Roem. & Schult.) DC. (Rubiaceae), *Vernonia* spp. (Asteraceae)	Sparse clinical data; mostly preliminary *in vivo* or ethnobotanical references

The category ‘clinical evidence available’ denotes the existence of published human clinical trial data for the species. It does not imply low risk of bias, high methodological quality, or sufficient clinical evidence to recommend routine use. Where the name listed in RENISUS, is a taxonomic synonym, the currently accepted name is given: *Libidibia ferrea* [syn. *Apuleia ferrea*], *Fridericia chica* [syn. *Arrabidaea chica*], *Eleutherine bulbosa* [syn. *Eleutherine plicata*], *Attalea speciosa* [syn. *Orbignya speciosa*], *Solidago chilensis* [syn. *Solidago microglossa*], *Monteverdia ilicifolia* [syn. *Maytenus ilicifolia*], *Lippia origanoides* [syn. *Lippia sidoides*], *Schinus terebinthifolia* [orth. var. *S. terebinthifolius*]. General abbreviations applicable to all tables: SUS, Sistema Único de Saúde; RENISUS, Relação Nacional de Plantas Medicinais de Interesse ao SUS; RCT, randomized controlled trial; OM, oral mucositis; RT, radiotherapy; QoL, quality of life; FITOPROT, mucoadhesive formulation containing curcuminoids from *Curcuma longa* L. (Zingiberaceae) and *Bidens pilosa* L. (Asteraceae) extract; HbA1c, glycated haemoglobin; n, number of participants; NR, not reported in the primary study. Botanical binomials are italicized; taxonomic authorities and family names are not. Accepted names follow World Flora Online (WFO) and Flora e Funga do Brasil (Reflora), as listed in Table 1.

**TABLE 3:** Human studies on the most frequently investigated RENISUS medicinal plants, with details of the tested preparation and dose regimen.

Species	Design	n	Condition	Plant part	Preparation tested	Dose/regimen	Standardization marker	Reference
*Ananas comosus*	RCT	50	Acute irritative cough (paediatric)	Fruit (bromelain)	Honey + bromelain oral syrup	NR	NR	Peixoto et al., 2016
*Ananas comosus*	Quasi-experimental cohort	100	Postpartum uterine involution	Fruit	Fresh fruit juice	200 mL/day	None (whole juice)	Batubara and Pulungan, 2022
*Ananas comosus*	Experimental (multiple extracts)	30	Uterine contractility	Fruit and stem	Aqueous and hydroalcoholic extracts	Concentration-dependent range	Partial (extract types described)	Monji et al., 2016
*Ananas comosus*	RCT, double-blind, placebo-controlled	60	Post-episiotomy wound healing	Stem and fruit (bromelain)	Oral bromelain tablet	500 mg every 8 h for 6 days	Reported (proteolytic activity)	Golezar, 2016
*Ananas comosus*	RCT	80	Metabolic health (overweight/obese women)	Fruit	Six-ingredient botanical beverage	200 mL/day; *A. comosus* proportion NR	NR	Dolores et al., 2018
*Ananas comosus*	Clinical pharmacokinetic study	40	Chronic rhinosinusitis	Fruit (bromelain)	Oral bromelain tablet	NR	NR	Passali et al., 2018
*Bidens pilosa*	Triple-blind RCT, placebo-controlled	120	Radiochemotherapy-induced OM	Aerial parts	FITOPROT mucoadhesive gel/rinse	5 mL, 3–4×/day throughout RT	Yes (curcuminoid content)	Arantes et al., 2021
*Bidens pilosa*	Pilot study	14	Glycemic regulation (men)	Whole plant	Oral formulation	NR	NR	Lai et al., 2015
*Bidens pilosa*	RCT, phase I	20	OM (oncology)	Aerial parts	FITOPROT mucoadhesive gel/rinse	Phase I dose escalation	Yes (curcuminoid content)	Santos Filho et al., 2018
*Bidens pilosa*	RCT	52	Radiotherapy-induced OM and QoL	Aerial parts	FITOPROT mucoadhesive gel/rinse	Per protocol, throughout RT	Yes (curcuminoid content)	Martins et al., 2023
*Copaifera officinalis*	Case series	6	Chronic wound healing	Trunk oleoresin	Topical oleoresin	NR	NR	Arroyo-Acevedo et al., 2011
*Copaifera langsdorffii*	Case report	1	Herb-induced hepatotoxicity	Trunk oil-resin	Oral, combined with *Hypericum perforatum*	NR	NR	Agollo et al., 2014
*Handroanthus impetiginosus*	Single-arm, open-label trial	12	Primary dysmenorrhea	Bark	Oral capsule (bark extract)	NR	Partial (naphthoquinones mentioned, not quantified)	McClure et al., 2022
*Handroanthus impetiginosus*	Case report	1	Oesophageal cancer	Bark	Oral extract, complementary therapy	NR	NR	Masaaki and Youichi, 2015
*Handroanthus impetiginosus*	Case report	1	Diffuse large B-cell lymphoma	Bark	Oral extract, complementary therapy	NR	NR	Hideto, 2017
*Handroanthus impetiginosus*	Case report	1	Metastatic thyroid cancer	Bark	Oral extract, complementary therapy	NR	NR	Hideto, 2016
*Anacardium occidentale*	RCT	40	Childhood malnutrition	Seed	Cashew nut seed flour, dietary	Dietary proportion NR	Not applicable (whole food)	Pereira de Jesus Costa et al., 2020
*Anacardium occidentale*	Clinical study	50	Oily skin (dermatology)	Bark/shell	Topical cosmetic formulation	NR	NR	Mercurio et al., 2017
*Anacardium occidentale*	Phase II clinical trial	30	Inflammatory wrist joint lesions	Stem bark	Topical cream, crude extract	Cream concentration NR	NR	Padilha et al., 2020
*Anacardium occidentale*	RCT	100	Cardiometabolic risk/appetite	Nut	Mixed-nut dietary intervention	30 g/day mixed nuts; *A. occidentale* proportion NR	NR	Rocha et al., 2023

NR, indicates that the primary study did not report the parameter; this reflects a reporting gap in the source literature rather than an omission in the present review. ‘Standardization marker: Yes’ indicates that the study explicitly quantified a marker compound; ‘Partial’ indicates qualitative mention without analytical verification. All studies listed involve human participants; case reports are included for safety signal documentation only. General abbreviations applicable to all tables: SUS, Sistema Único de Saúde; RENISUS, Relação Nacional de Plantas Medicinais de Interesse ao SUS; RCT, randomized controlled trial; OM, oral mucositis; RT, radiotherapy; QoL, quality of life; FITOPROT, mucoadhesive formulation containing curcuminoids from *Curcuma longa* L. (Zingiberaceae) and *Bidens pilosa* L. (Asteraceae) extract; HbA1c, glycated haemoglobin; n, number of participants; NR, not reported in the primary study. Botanical binomials are italicized; taxonomic authorities and family names are not. Accepted names follow World Flora Online (WFO) and Flora e Funga do Brasil (Reflora), as listed in Table 1.

The following sections provide an in-depth look at the current evidence for *A. comosus*, *B. pilosa*, *Copaifera* spp., *H. impetiginosus*, and *A. occidentale*.

### 3.3 Ananas comosus (L.) Merr

*Ananas comosus* (L.) Merr. (Bromeliaceae), commonly referred to as pineapple, has been investigated in multiple clinical contexts, including women’s health, ear, nose, and throat (ENT) conditions, and post-surgical recovery (Golezar, 2016; Batubara and Pulungan, 2022; Passali et al., 2018). Research predominantly centers on bromelain, the plant’s proteolytic enzyme complex, which is recognized for its anti-inflammatory and tissue-healing properties (Peixoto et al., 2016; Monji et al., 2016).

A combination of honey and a bromelain-containing syrup demonstrated no measurable effect on acute irritative cough in 50 children; Peixoto et al. (2016) reported no significant difference compared to controls. This finding is frequently overlooked in reviews of bromelain research.

Studies on postpartum recovery yielded notable findings. In a cohort of 100 mothers receiving 200 mL of fresh pineapple fruit juice daily, pineapple juice accelerated uterine involution compared to controls (Batubara and Pulungan, 2022). Another study involving 30 participants utilized aqueous and hydroalcoholic fruit and stem extracts instead of juice, tested across a concentration-dependent range, and reported effects on uterine contractility (Monji et al., 2016), suggesting a possible uterotonic mechanism. However, the small sample size limits the strength of these conclusions.

In a randomized, double-blind, placebo-controlled trial involving 60 women recovering from episiotomy, oral bromelain (500 mg every 8 h for 6 days, derived from stem and fruit) reduced perineal pain and accelerated wound closure relative to placebo (Golezar, 2016).

For clinical application, bromelain must reach the target tissue. Passali et al. (2018) directly assessed this in 40 patients with chronic rhinosinusitis receiving oral bromelain tablets and detected measurable concentrations in both the bloodstream and sinonasal mucosa.

A metabolic study included pineapple fruit as one ingredient of a six-ingredient beverage (200 mL/day), which also contained *Physalis peruviana* L. (Solanaceae), *Passiflora edulis* Sims (Passifloraceae), *Avena sativa* L. (Poaceae), *Linum usitatissimum* L. (Linaceae), and *Stevia rebaudiana* (Bertoni) Bertoni (Asteraceae), administered to 80 overweight and obese women (Dolores et al., 2018). Improvements were observed in lipid profile and glycemia; however, the study design did not allow attribution of these changes to any specific ingredient (Table 4).

**TABLE 4:** Clinical studies on Ananas comosus (L.) Merr. (Bromeliaceae).

Study	Plant part used	n	Research findings
Use of honey associated with *Ananas comosus* (bromelain) in the treatment of acute irritative cough (Peixoto et al., 2016)	Fruit (bromelain)	50	The combination of honey and bromelain showed no significant difference in treating acute irritative cough compared to the control group
Multimodal care for headaches, lumbopelvic pain, and dysmenorrhea in a woman with endometriosis: a case report (Martin, 2021)	Not specified	1	The case report did not specifically focus on *A. comosus* but discussed multimodal treatment for endometriosis-related pain, including approaches that may involve bromelain or pineapple juice
The effect of pineapple juice (*Ananas comosus*) on uterine involution in postpartum mothers (Batubara and Pulungan, 2022)	Fruit juice	100	Pineapple juice consumption (200 mL/day) accelerated uterine involution in postpartum mothers, leading to faster uterine size reduction compared to the control group
Investigation of uterotonic properties of *Ananas comosus* extracts (Monji et al., 2016)	Fruit and stem (multiple extracts)	30	*A. comosus* extracts demonstrated uterotonic effects across a concentration-dependent range, contributing to more efficient uterine activity
*Ananas comosus* effect on perineal pain and wound healing after episiotomy: a randomized double-blind placebo-controlled clinical trial (Golezar, 2016)	Stem and fruit (bromelain)	60	Oral bromelain (500 mg every 8 h for 6 days) accelerated perineal wound healing and significantly reduced post-episiotomy pain compared to placebo
Effect of a beverage containing *Physalis peruviana*, *Passiflora edulis*, *Ananas comosus*, *Avena sativa*, *Linum usitatissimum*, and *Stevia rebaudiana* on lipid profile and glycemia (Dolores et al., 2018)	Fruit (in plant mixture)	80	The beverage (200 mL/day) led to improvements in lipid profile and blood glucose in overweight and obese women. Effects cannot be attributed to any single ingredient
Bromelain’s penetration into the blood and sinonasal mucosa in patients with chronic rhinosinusitis (Passali et al., 2018)	Fruit (bromelain)	40	Bromelain effectively penetrated the sinonasal mucosa and bloodstream, providing relief from chronic rhinosinusitis symptoms

General abbreviations applicable to all tables: SUS, Sistema Único de Saúde; RENISUS, Relação Nacional de Plantas Medicinais de Interesse ao SUS; RCT, randomized controlled trial; OM, oral mucositis; RT, radiotherapy; QoL, quality of life; FITOPROT, mucoadhesive formulation containing curcuminoids from *Curcuma longa* L. (Zingiberaceae) and *Bidens pilosa* L. (Asteraceae) extract; HbA1c, glycated haemoglobin; n, number of participants; NR, not reported in the primary study. Botanical binomials are italicized; taxonomic authorities and family names are not. Accepted names follow World Flora Online (WFO) and Flora e Funga do Brasil (Reflora), as listed in Table 1.

### 3.4 Bidens pilosa L. (Asteraceae)

Three randomized trials have investigated *B. pilosa* L. (Asteraceae) in a single clinical context: oral mucositis in patients undergoing radiochemotherapy. This specificity enhances the interpretability of the evidence, as it is concentrated on one application rather than dispersed across multiple conditions.

The formulation evaluated in these trials consists of *B. pilosa* aerial-part hydroethanolic extract combined with curcuminoids from *Curcuma longa* L. (Zingiberaceae) in a mucoadhesive vehicle known as FITOPROT. The product is standardized to its curcuminoid content and administered as a 5 mL oral rinse or gel applied three to four times daily throughout the radiotherapy course. Arantes et al. (2021) conducted a triple-blind, placebo-controlled trial with 120 patients and observed reduced mucositis severity from day 15 onward, as well as fewer cases of ulcerative lesions by the end of radiotherapy. Santos Filho et al. (2018) conducted a phase I dose-escalation study with 20 patients and reported transient mucosal pigmentation and tooth sensitivity as side effects; both resolved after discontinuation, and no genotoxic effects were observed.

Martins et al. (2023) monitored 52 patients receiving the same formulation and found no statistically significant differences between groups regarding the primary endpoint. Both groups experienced a decline in quality of life during radiotherapy. In the trials where these biomarkers were measured, IL-8 levels and salivary nitrite decreased in the treatment group (Santos Filho et al., 2018; Martins et al., 2023). A separate pilot study administered an oral *B. pilosa* whole-plant formulation to 14 men and reported improved glycemic control and insulin secretion, although the preparation and dose were not fully characterized (Lai et al., 2015). Table 5 presents the study designs, sample sizes, primary endpoints, and biomarker findings for these trials.

**TABLE 5:** Clinical trials on Bidens pilosa L. (Asteraceae).

Study	n	Study type	Findings
Safety and efficacy of a mucoadhesive phytomedication containing curcuminoids and *Bidens pilosa* in the prevention and treatment of oral mucositis (Arantes et al., 2021)	120	Triple-blind, randomized, placebo-controlled clinical trial	FITOPROT reduced OM severity from day 15 until the end of RT, while placebo showed increased severity (p < 0.05). The intervention group had fewer patients with ulcerative OM at the end of RT (p < 0.05). The formulation prevented increases in IL-8 levels and reduced salivary nitrite during RT.
*Bidens pilosa* formulation improves blood homeostasis and β-cell function in men: a pilot study (Lai et al., 2015)	14	Pilot study	Improved blood glucose regulation and increased insulin secretion, suggesting benefits for glycemic control and β-cell function in men. Preparation and dose were not fully characterized
Effects of a mucoadhesive phytomedicine (*Curcuma longa* and *Bidens pilosa*) on radiotherapy-induced oral mucositis (Martins et al., 2023)	52	Randomized clinical trial	No statistical differences were found between groups for OM assessment. Both groups showed worsened quality of life during RT. No statistically significant differences were observed between groups for any of the evaluated measures
Randomized clinical trial of a mucoadhesive formulation containing curcuminoids (Zingiberaceae) and *Bidens pilosa* Linn (Asteraceae) extract (FITOPROT): phase I study (Santos Filho et al., 2018)	20	Randomized clinical trial, phase I	Observed side effects included temporary, low-intensity mucosal/dental surface pigmentation (n = 7) and tooth sensitivity (n = 4), which resolved after discontinuation. No significant cellular genotoxic effects were observed (p > 0.05), and micronucleus frequencies remained unchanged (p > 0.05)

The included studies employ diverse designs, with varying doses, comparators, and endpoints; therefore, direct comparison of results is not feasible. FITOPROT, is a multi-ingredient mucoadhesive formulation containing curcuminoids from *Curcuma longa* L. (Zingiberaceae) and *Bidens pilosa* L. (Asteraceae) extract, standardized to curcuminoid content and applied as a 5 mL rinse or gel 3–4 times daily throughout radiotherapy; observed effects cannot be attributed to *B. pilosa* alone. Reported adverse effects resolved following discontinuation of treatment. These summaries are descriptive and do not constitute clinical guidance. General abbreviations applicable to all tables: SUS, Sistema Único de Saúde; RENISUS, Relação Nacional de Plantas Medicinais de Interesse ao SUS; RCT, randomized controlled trial; OM, oral mucositis; RT, radiotherapy; QoL, quality of life; FITOPROT, mucoadhesive formulation containing curcuminoids from *Curcuma longa* L. (Zingiberaceae) and *Bidens pilosa* L. (Asteraceae) extract; HbA1c, glycated haemoglobin; n, number of participants; NR, not reported in the primary study. Botanical binomials are italicized; taxonomic authorities and family names are not. Accepted names follow World Flora Online (WFO) and Flora e Funga do Brasil (Reflora), as listed in Table 1.

### 3.5 Copaifera langsdorffii Desf. and Copaifera officinalis (Jacq.) L. (Fabaceae)

Copaiba oleoresin, obtained from the trunk of *Copaifera langsdorffii* Desf. and *Copaifera officinalis* (Jacq.) L. (Fabaceae), has a longstanding history of traditional use in wound treatment, and recent clinical studies provide evidence supporting its efficacy. For example, Leite et al. (2023) demonstrated that copaiba-based topical treatments accelerated healing and promoted tissue regeneration in patients with chronic wounds. Arroyo-Acevedo et al. (2011) reported comparable results in six patients treated with topically applied oleoresin, although neither study specified the oleoresin concentration or the volume applied per dressing.

However, safety concerns have been identified. Agollo et al. (2014) reported a case of liver toxicity associated with the oral use of *C. langsdorffii* oleoresin in combination with *Hypericum perforatum* L. (Hypericaceae) (St. John’s Wort), underscoring the potential risks of polyherbal products. This case indicates that combining copaiba with other botanicals or pharmaceuticals may increase hepatotoxic risk, which warrants clinical monitoring.

Table 6 summarizes clinical and observational studies on *Copaifera* species, specifying the study design, plant part utilized, number of participants, and principal findings. This compilation aims to provide a comprehensive overview of efficacy signals and reported adverse events (Arroyo-Acevedo et al., 2011; Agollo et al., 2014).

**TABLE 6:** Clinical and observational studies on Copaifera spp. (Fabaceae).

Study	Plant part used	n	Results
Healing of chronic wounds treated with copaiba oleoresin (Arroyo-Acevedo et al., 2011)	Oleoresin extracted from the trunk of *Copaifera officinalis* (Jacq.) L	6	Significant acceleration of chronic wound healing, demonstrating the therapeutic potential of the oleoresin. Concentration and volume applied were not reported
Hepatotoxicity induced by *Hypericum perforatum* with possible association with copaiba (Agollo et al., 2014)	Oil-resin harvested from the trunk of *Copaifera langsdorffii* Desf	1	Case report of hepatotoxicity, suggesting a herb–drug interaction between *H. perforatum* and *C. langsdorffii*, highlighting the importance of further safety studies

General abbreviations applicable to all tables: SUS, Sistema Único de Saúde; RENISUS, Relação Nacional de Plantas Medicinais de Interesse ao SUS; RCT, randomized controlled trial; OM, oral mucositis; RT, radiotherapy; QoL, quality of life; FITOPROT, mucoadhesive formulation containing curcuminoids from *Curcuma longa* L. (Zingiberaceae) and *Bidens pilosa* L. (Asteraceae) extract; HbA1c, glycated haemoglobin; n, number of participants; NR, not reported in the primary study. Botanical binomials are italicized; taxonomic authorities and family names are not. Accepted names follow World Flora Online (WFO) and Flora e Funga do Brasil (Reflora), as listed in Table 1.

### 3.6 Handroanthus impetiginosus (Mart. ex DC.) Mattos (bignoniaceae)

*Handroanthus impetiginosus* (Mart. ex DC.) Mattos (Bignoniaceae) [syn. *Tabebuia avellanedae* Lorentz ex Griseb.], known as pau d’arco or lapacho, has been explored in oncology and gynecology. McClure et al. (2022) found an oral bark-extract capsule preparation safe and tolerable in 12 adults with primary dysmenorrhea, although the naphthoquinone content of the preparation was not quantified. In addition to gynecological uses, case reports employing orally administered bark extracts as complementary therapy suggest that pau d’arco may have a supportive role in cancer care, with observed benefits in esophageal cancer, lymphoma, and metastatic thyroid cancer (Masaaki and Youichi, 2015; Hideto, 2016; Hideto, 2017).

Table 7 provides a summary of the clinical and observational studies conducted on *H. impetiginosus*.

**TABLE 7:** Clinical and observational studies on Handroanthus impetiginosus (Mart. ex DC.) Mattos (Bignoniaceae).

Study	Plant part used	n	Research findings
Safety and tolerability of Pau d'Arco (*Handroanthus impetiginosus*) for primary dysmenorrhea: a single-arm, open-label trial on adults ages 18–45 (McClure et al., 2022)	Bark extract (oral capsule)	12	Pau d’arco was safe and well-tolerated, with significant reduction in dysmenorrhea symptoms among participants. Naphthoquinone content was not quantified
*Handroanthus impetiginosus* and oesophageal cancer: a case report (Masaaki and Youichi, 2015)	Bark extract (oral)	1	Case report describing a positive response in an oesophageal cancer patient using complementary treatment
*Handroanthus impetiginosus* and diffuse large B-cell lymphoma: a case report (Hideto, 2017)	Bark extract (oral)	1	Case report suggesting potential as adjuvant therapy for B-cell lymphoma, with observed patient improvement
Metastatic thyroid cancer treated with *Handroanthus impetiginosus*: a first case report (Hideto, 2016)	Bark extract (oral)	1	First reported case of use in metastatic thyroid cancer, indicating positive disease control response

Pau d’arco bark extract may be obtained from different bark portions and solvents, with variable naphthoquinone content and doses. Oncology cases used the extract as complementary therapy. The four studies cite the species under its synonym *Tabebuia avellanedae* Lorentz ex Griseb.; the currently accepted name is used here. General abbreviations applicable to all tables: SUS, Sistema Único de Saúde; RENISUS, Relação Nacional de Plantas Medicinais de Interesse ao SUS; RCT, randomized controlled trial; OM, oral mucositis; RT, radiotherapy; QoL, quality of life; FITOPROT, mucoadhesive formulation containing curcuminoids from *Curcuma longa* L. (Zingiberaceae) and *Bidens pilosa* L. (Asteraceae) extract; HbA1c, glycated haemoglobin; n, number of participants; NR, not reported in the primary study. Botanical binomials are italicized; taxonomic authorities and family names are not. Accepted names follow World Flora Online (WFO) and Flora e Funga do Brasil (Reflora), as listed in Table 1.

### 3.7 Anacardium occidentale L. (Anacardiaceae)

The cashew tree, *A. occidentale* L. (Anacardiaceae), has demonstrated promising outcomes in metabolic health and nutritional interventions. Clinical trials involving malnourished children reported that cashew nut seed flour, incorporated into the daily diet, was associated with improvements in glycated hemoglobin (HbA1c) and lipid profiles, indicating its potential role in addressing childhood malnutrition (Pereira de Jesus Costa et al., 2020).

In addition to its nutritional value, the plant’s bioactive metabolites, particularly anacardic acids, have demonstrated therapeutic potential (Mercurio et al., 2017). A phase II trial applied a topical cream containing crude stem-bark extract to 30 patients with inflammatory wrist joint lesions and reported a significant reduction in inflammatory symptoms, although the extract concentration in the cream was not reported (Padilha et al., 2020). A further randomized trial administered 30 g/day of a mixed-nut preparation containing *A. occidentale* to 100 women at cardiometabolic risk and observed reduced hunger, though the proportion attributable to cashew was not isolated (Rocha et al., 2023). Table 8 provides a comprehensive summary of key studies on *A. occidentale*.

**TABLE 8:** Clinical studies on Anacardium occidentale L. (Anacardiaceae).

Study	Plant part used	n	Research findings
Effects of cashew nut (*Anacardium occidentale* L.) seed flour in moderately malnourished children: randomized clinical trial (Pereira de Jesus Costa et al., 2020)	Seed (cashew nut flour)	40	Cashew nut seed flour improved nutritional profile and aided recovery of moderately malnourished children, with improvements in HbA1c and lipid profile
*Anacardium occidentale* L. extract in cosmetic formulations: benefits for oily skin (Mercurio et al., 2017)	Bark/shell extract (topical)	50	Extract demonstrated benefits for oily skin in cosmetic formulations, with positive effects on reducing oiliness and improving overall skin appearance
Alpha-amylase inhibition and antioxidative capacity of some antidiabetic plants used by traditional healers in Southeastern Nigeria (Oyedemi et al., 2017)	Traditional antidiabetic plants (*in vitro*)	Not applicable	Plants showed alpha-amylase inhibition and antioxidant capacity *in vitro*. No clinical outcomes in humans were reported for *A. occidentale*
Phase II clinical pharmacological trial of a cream containing crude extract from *Anacardium occidentale* L. bark in the treatment of inflammatory wrist joint lesions (Padilha et al., 2020)	Stem bark (crude extract, topical cream)	30	The cream was effective in treating inflammatory wrist joint lesions, with significant reduction in inflammatory symptoms. Extract concentration in the cream was not reported
Nut-enriched energy-restricted diet has potential to decrease hunger in women at cardiometabolic risk: a randomized controlled trial (Rocha et al., 2023)	Nut (in mixed-nut preparation)	100	The energy-restricted diet enriched with Brazilian nuts (30 g/day) reduced hunger in women at cardiometabolic risk. The proportion attributable to *A. occidentale* was not isolated

General abbreviations applicable to all tables: SUS, Sistema Único de Saúde; RENISUS, Relação Nacional de Plantas Medicinais de Interesse ao SUS; RCT, randomized controlled trial; OM, oral mucositis; RT, radiotherapy; QoL, quality of life; FITOPROT, mucoadhesive formulation containing curcuminoids from *Curcuma longa* L. (Zingiberaceae) and *Bidens pilosa* L. (Asteraceae) extract; HbA1c, glycated haemoglobin; n, number of participants; NR, not reported in the primary study. Botanical binomials are italicized; taxonomic authorities and family names are not. Accepted names follow World Flora Online (WFO) and Flora e Funga do Brasil (Reflora), as listed in Table 1.

The interventions assessed, such as seed-flour diet, topical bark-extract cream, cosmetic formulation, and mixed-nut diet, vary considerably, which restricts direct comparability of outcomes. Observed effects in mixed-nut or multi-metabolite products cannot be ascribed exclusively to *A. occidentale*. Furthermore, the cited study on traditional antidiabetic plants provides only *in vitro* enzyme and antioxidant activity data, without reporting clinical outcomes in humans for *A. occidentale* (Oyedemi et al., 2017).

The reviewed literature demonstrates the broad therapeutic potential of *A. comosus*, *B. pilosa*, *Copaifera* spp., *H. impetiginosus*, and *A. occidentale*. These species have shown promising results in pain relief, wound healing, cancer care, and metabolic support. However, additional rigorous clinical trials are necessary to confirm these benefits and to establish clear, evidence-based guidelines for their safe and effective use.

### 3.8 Critical methodological appraisal of clinical evidence

While the five prioritized species (*A. comosus*, *B. pilosa*, *Copaifera* spp., *H. impetiginosus*, and *A. occidentale*) show promise, a thorough evaluation of their clinical studies reveals high heterogeneity that challenges generalizability—spanning from wound healing and oral mucositis to metabolic management in obesity. Furthermore, some of the reviewed clinical trials exhibit major methodological weaknesses:

- Sample size and power: small cohorts (e.g., n = 30 to n = 100 subjects) predominate, drastically limiting the statistical power required to confirm subtle systemic therapeutic advantages.
- Randomization and blinding: several trial models fail to thoroughly document randomization sequences or double-blind procedures, leaving data vulnerable to investigator bias.
- Dosing regimens and characterization: a critical flaw across the literature is the reporting of pharmacological actions where the chemical profile of the extract (e.g., specific solvent-to-solid ratios or standardized marker metabolite yields) is omitted. As shown in Table 3, only the FITOPROT trials report a standardization marker; the remaining studies characterize neither the extract nor, in several cases, the administered dose.
- Model relevance: many claims rely excessively on *in vitro* assays (such as free-radical scavenging) that cannot predict complex *in vivo* anti-inflammatory pathways or systemic clinical bioavailability within the human public health setting (Almada-Vilhena et al., 2024).

## 4 Discussion

### 4.1 Anti-inflammatory and antioxidant properties

Anti-inflammatory and antioxidant effects represent some of the most extensively documented properties of RENISUS species. *Bidens pilosa* is frequently cited for these effects, with studies demonstrating that its extracts reduce edema, downregulate cytokine expression, and attenuate oxidative stress in both acute and chronic inflammation models. The molecular mechanisms underlying these outcomes are commonly attributed to polyacetylenes, caffeic acid derivatives, and polysaccharides (Allegra, 2019).

Oleoresins derived from *Copaifera* species, particularly *C. langsdorffii*, exhibit potent anti-inflammatory activity. Experimental studies report modulation of immune cell infiltration, inhibition of cyclooxygenase pathways, and suppression of nitric oxide production (Boccolini and Boccolini, 2020).

Bromelain, a proteolytic enzyme complex extracted from *A. comosus*, is another extensively studied anti-inflammatory agent. Clinical studies report reductions in edema and pain, as well as shorter healing times following surgery, with applications in oral, musculoskeletal, and gynecological settings (Batubara and Pulungan, 2022; Passali et al., 2018).

Other RENISUS species, including *Eugenia uniflora* L. (Myrtaceae), *Monteverdia ilicifolia* (Mart. ex Reissek) Biral (Celastraceae) [syn. *Maytenus ilicifolia* Mart. ex Reissek], *Baccharis trimera* (Less.) DC. (Asteraceae), and *Lippia origanoides* Kunth (Verbenaceae) [syn. *Lippia sidoides* Cham.], are frequently cited for their anti-inflammatory and antioxidant effects (Martins et al., 2023; Lima Melro et al., 2020; Santos et al., 2011). Numerous studies underscore their significance in modulating oxidative stress associated with metabolic and degenerative diseases.

Research on *Casearia sylvestris* Sw. (Salicaceae) consistently demonstrates its potent anti-inflammatory and antioxidant effects. *In vitro* and animal studies indicate that this plant inhibits key pro-inflammatory mediators and efficiently scavenges free radicals. These effects are primarily attributed to its phenolic metabolites and flavonoids, which are commonly identified as the principal bioactive constituents.

### 4.2 Wound healing and tissue repair

Wound healing represents a significant area of investigation for several RENISUS plants, particularly *C. sylvestris*, *Copaifera* species, and *A. comosus*. Extracts of *C. sylvestris* have demonstrated the ability to accelerate wound contraction and facilitate re-epithelialization while reducing inflammation. Further studies indicate increased granulation tissue, enhanced angiogenesis, and improved tensile strength of healed tissue.

*Copaifera* oleoresin, which is widely utilized in traditional medicine, has been supported by recent studies demonstrating its promotion of granulation tissue formation, reduction of infection risk, and acceleration of healing in chronic ulcers. However, certain clinical observations have reported hepatotoxicity when *Copaifera* is administered in combination with other botanical drugs, underscoring the necessity for careful monitoring.

The active enzyme bromelain, derived from *A. comosus*, has been associated with reduced inflammation, alleviation of perineal pain following childbirth, and facilitation of uterine involution. Clinical trials support its application in gynecological and oral procedures.

Additional plants frequently cited for their wound-healing properties include *F. chica* (Bonpl.) L.G.Lohmann (Bignoniaceae) [syn. *Arrabidaea chica* (Bonpl.) Verl.] and *Schinus terebinthifolia* Raddi (Anacardiaceae). *F. chica*, in particular, is extensively utilized in traditional medicine for the treatment of burns and skin ulcers.

### 4.3 Antitumor and chemopreventive properties

A systematic review of Brazilian Cerrado plants identified antitumor activity in tumor cell lines, highlighting preclinical research opportunities rather than established clinical efficacy (Oliveira et al., 2024). Research on selected Brazilian plant species has investigated cytotoxic and chemopreventive potential alongside toxicological effects, underscoring the need to evaluate biological activity and safety together (Silva et al., 2020).

*Bidens pilosa* has demonstrated significant potential in clinical research addressing oral mucositis among cancer patients. Randomized trials indicate that mucoadhesive formulations containing *B. pilosa* reduce ulceration and pain during radiochemotherapy (Arantes et al., 2021; Santos Filho et al., 2018). In addition to clinical applications, laboratory studies have identified cytotoxic effects of *B. pilosa* extracts against various tumor cell lines.

*Handroanthus impetiginosus* exhibits cytotoxic activity in cancer models, with notable effects observed in lymphoma. Open-label studies and case reports document symptom improvement and indicate a potential role as an adjunct therapy (McClure et al., 2022; Hideto, 2017).

*Casearia sylvestris* and *Copaifera* species are frequently recognized for their chemopreventive and cytotoxic properties. An increasing body of preclinical research demonstrates their capacity to inhibit tumor growth and modulate pathways involved in cancer development.

Evidence indicates that *A. occidentale* can sensitize tumor cells to chemotherapy. Additionally, it has been shown to inhibit alpha-amylase and affect multiple biological pathways implicated in cancer progression (Mercurio et al., 2017).

### 4.4 Metabolic and antidiabetic activity

Metabolic and nutritional research is particularly abundant for *A. occidentale* and *B. pilosa*. In children, clinical work with cashew-nut flour reports improvements in glycemic and lipid profiles, better recovery from malnutrition, and a reduced risk for diabetes (Pereira de Jesus Costa et al., 2020; Mercurio et al., 2017).

Supplementation with *B. pilosa* is supported by pilot trials for glycemic control and insulin secretion, suggesting benefits for glucose homeostasis (Lai et al., 2015). Other species, such as *M. ilicifolia*, are under study for gastroprotective and antidiabetic effects (Dolores et al., 2018; Martins et al., 2023).

### 4.5 Antimicrobial, antiparasitic, and antifungal activity

Extensive research demonstrates the antimicrobial properties of *C. sylvestris*, *B. pilosa*, and *Copaifera* species, with documented activity against a range of bacteria, fungi, and protozoa. *Lippia origanoides* and *S. terebinthifolia* have demonstrated notable efficacy in the treatment of infections affecting the genitourinary tract, skin, and oral cavity.

The therapeutic versatility of RENISUS species is further evidenced by the use of *Costus spiralis* for kidney protection, *B. trimera* for liver health, and *S. terebinthifolia* and *Stryphnodendron adstringens* (Mart.) Coville (Fabaceae) in the management of gynecological and urinary conditions.

Within the RENISUS plants reviewed, *A. comosus* and *B. pilosa* are distinguished by consistent evidence supporting their roles in inflammation, wound healing, and metabolic health. *Copaifera* species and *H. impetiginosus* exhibit more specific potential in wound care and oncology, respectively, whereas *A. occidentale* demonstrates value in applications that integrate nutrition and metabolic regulation.

A significant barrier to clinical application is the absence of standardized preparations, dosing guidelines, and extraction methodologies. Variability in plant parts, solvent systems, and extract concentrations complicates the comparison of results across studies and hinders reproducibility. The development of pharmacopeial standards and validated production protocols is essential for integrating traditional knowledge with contemporary evidence-based healthcare.

### 4.6 Safety profile and adverse events

Bromelain is generally well-tolerated in short-term use. Reported adverse effects include mild gastrointestinal upset and hypersensitivity reactions, with cross-reactivity documented in patients allergic to latex or pineapple. Given its documented uterotonic properties (Monji et al., 2016; Batubara and Pulungan, 2022), caution is warranted during pregnancy and the postpartum period beyond supervised clinical protocols. Paediatric data are limited to the cough trial reported by Peixoto et al. (2016), which identified no safety signals in 50 children. No long-term toxicity data are available from the reviewed literature.

The FITOPROT formulation demonstrated a favourable short-term safety profile in reviewed trials. Santos Filho et al. (2018) reported transient mucosal pigmentation and tooth sensitivity as the sole adverse effects, both resolving after discontinuation, with no genotoxic signal. No contraindication data specific to pregnancy, lactation, or paediatric populations were identified in the reviewed clinical literature for *B. pilosa*.

Hepatotoxicity represents the most significant safety concern identified for *Copaifera* species. Agollo et al. (2014) reported a case of herb-induced liver injury associated with the co-administration of *C. langsdorffii* and *H. perforatum*, consistent with a CYP450-mediated herb–drug interaction. Self-administration of oleoresin in quantities exceeding therapeutic doses has been associated with gastrointestinal disturbances in isolated reports. The use of *Copaifera* oleoresin during pregnancy and in paediatric populations is not supported by controlled clinical evidence.

*Handroanthus impetiginosus* was reported as safe and well-tolerated at the doses tested in the dysmenorrhea trial (McClure et al., 2022). However, the naphthoquinone content of pau d’arco preparations varies substantially depending on bark provenance, solvent, and extraction conditions, introducing unpredictable pharmacological variability and potential dose-related toxicity risk. No controlled data addressing contraindications in pregnancy, lactation, or paediatric use were identified.

Cashew preparations were well-tolerated in the reviewed clinical trials. Known allergy risk exists in patients with tree-nut hypersensitivity; cross-reactivity with urushiol-containing plants has been documented. Cashew bark extracts contain anacardic acids, which carry potential dermatological irritancy, particularly in topical formulations (Padilha et al., 2020). Long-term safety data and controlled evidence for paediatric or pregnant populations are lacking.

### 4.7 Clinical implications and policy recommendations

For clinicians working within the Farmácia Viva program or Basic Health Units, the evidence reviewed indicates that *B. pilosa* (in the FITOPROT formulation) and bromelain from *A. comosus* present the most methodologically consistent signals, for oral mucositis and gynecological applications respectively. These species may be considered for inclusion in institutional therapeutic protocols under medical supervision, with mandatory communication and monitoring of documented herb–drug interactions and hepatotoxic risk signals.

For researchers, this review highlights a critical evidence gap: of 71 RENISUS species, only five have undergone human clinical investigation, and that body of evidence is characterized by substantial methodological variability. Priority research areas include standardized extract characterization, pharmacovigilance integration, and herb–drug interaction profiling, particularly for species already in common use in SUS settings or available over the counter.

For policymakers, a tiered regulatory approach appears warranted. Species with convergent preclinical and clinical support (e.g., *B. pilosa* for mucositis) could be prioritized for accelerated inclusion pathways, while species associated with safety signals (e.g., *Copaifera* spp. in combination with other botanicals) should be subject to mandatory pharmacovigilance protocols before broader institutional adoption. Nevertheless, local implementation remains challenging, particularly in integrating medicinal plants into oral healthcare and coordinating the relevant public policies (Teixeira, Conceição, 2023).

### 4.8 Limitations

This review presents operational limitations. The protocol was not prospectively registered in PROSPERO, and standardized risk-of-bias evaluation tools (e.g., Cochrane RoB two or ROBINS-I) were not applied. These choices reflect the scoping design focused on establishing a baseline inventory for the 71 RENISUS species rather than evaluating comparative clinical efficacy.

Caution is warranted when interpreting trials involving multi-ingredient formulations, such as *B. pilosa* evaluated within FITOPROT, *A. comosus* in multi-component botanical beverages, and *Copaifera* species co-administered with *H. perforatum*. In these polyherbal interventions, observed therapeutic efficacy or adverse events cannot be definitively attributed solely to the target RENISUS species, as synergistic, additive, or confounding constituent interactions remain unisolated.

A further limitation concerns the primary literature itself. As Table 3 documents, most included clinical studies do not report a standardization marker for the tested preparation, and several omit the administered dose entirely. This reflects a reporting gap in the source studies rather than an omission in the present synthesis, but it constrains the extent to which dose–response relationships can be inferred. Future review updates could evaluate automation tools to support selected stages of the review process, with explicit reporting of their use and human oversight (Van Dinter et al., 2021).

## 5 Conclusion

This systematic review mapped and critically examined the scientific evidence for the 71 medicinal plants included in the Brazilian National List of Medicinal Plants of Interest to the Unified Health System (RENISUS). For most species, available data were restricted to *in vitro* assays and *in vivo* preclinical studies, with limited toxicological investigations and minimal clinical evaluation. The resulting descriptive classification, which distinguishes species by low, moderate, or clinical-evidence-available levels, offers a structured framework to identify research gaps and establish priorities for future studies and for the rational incorporation of phytotherapeutic options into the Unified Health System (SUS).

Among the species reviewed, five were identified as particularly relevant due to the availability of human clinical data: *A. occidentale*, *A. comosus*, *B. pilosa*, *Copaifera* spp., and *H. impetiginosus*. Synthesis of these studies demonstrated evidence of efficacy in clinically significant areas, including wound healing and tissue repair, management of oral mucositis in oncology, gynecological conditions such as dysmenorrhea and postpartum uterine involution, metabolic regulation, and supportive care in oncological contexts. These findings are consistent with traditional uses reported for these species and indicate that, under appropriate conditions, they may contribute to expanding therapeutic options in both primary and specialized care. Nevertheless, reports of hepatotoxicity, acute kidney injury, and potential herb–drug interactions, along with methodological limitations in several trials, highlight the importance of jointly considering efficacy and safety in scientific validation. Uncritical dissemination of these products could result in preventable harm.

The review also identified structural limitations within the current evidence base. Variability in study designs, small sample sizes, lack of standardized preparations, and insufficient reporting of adverse events limited the feasibility of quantitative synthesis and impeded extrapolation of results across populations and clinical contexts. The absence of a validated risk-of-bias assessment tool precludes formal quality grading of included studies and may have influenced the interpretation of findings by preventing systematic identification and weighting of high-risk studies. Future systematic reviews on RENISUS species should integrate validated instruments such as Cochrane RoB 2.0 for randomized trials and ROBINS-I for observational studies. Many RENISUS species are supported primarily by ethnobotanical information or isolated experimental studies, with minimal toxicological characterization. Addressing these gaps by strengthening toxicology, pharmacovigilance, and herb–drug interaction studies, particularly for species already used in SUS or available over the counter, is essential to prevent perpetuating disparities in access, information, and risk observed with other therapeutic technologies.

These findings indicate that RENISUS should be regarded not merely as a technical inventory of resources, but as a dynamic interface connecting traditional knowledge systems, biodiversity governance, and public health policies. Advancements in scientific validation and the integration of these plants into the Unified Health System (SUS) require rigorous clinical and toxicological studies, as well as research strategies that acknowledge traditional knowledge holders as co-producers of evidence, ensure equitable benefit-sharing, and prevent new forms of epistemic and economic extraction. In practice, this necessitates the promotion of community-engaged research, the strengthening of programs such as Farmácia Viva, and the alignment of phytotherapy policies with broader frameworks addressing biodiversity, human rights, and health equity.

This study offers a systematic mapping of research, a descriptive classification of evidence levels, and a critical analysis of five clinically studied species, thereby providing a foundation for decision-making in research, regulation, and health policy. Future investigations that integrate experimental and clinical designs with participatory approaches will be essential for the safe and equitable incorporation of these plants into the Unified Health System (SUS).
