# Supplementary material for: Traditional medicinal plants in the Unified Health System of Brazil: a systematic review
Source: Front Pharmacol. 2026 Oct 21;17:1867902. doi: 10.3389/fphar.2026.1867902 (PMC13731864; doi:10.3389/fphar.2026.1867902)
Supplement: Supplementary file 1 [file DataSheet1.pdf]

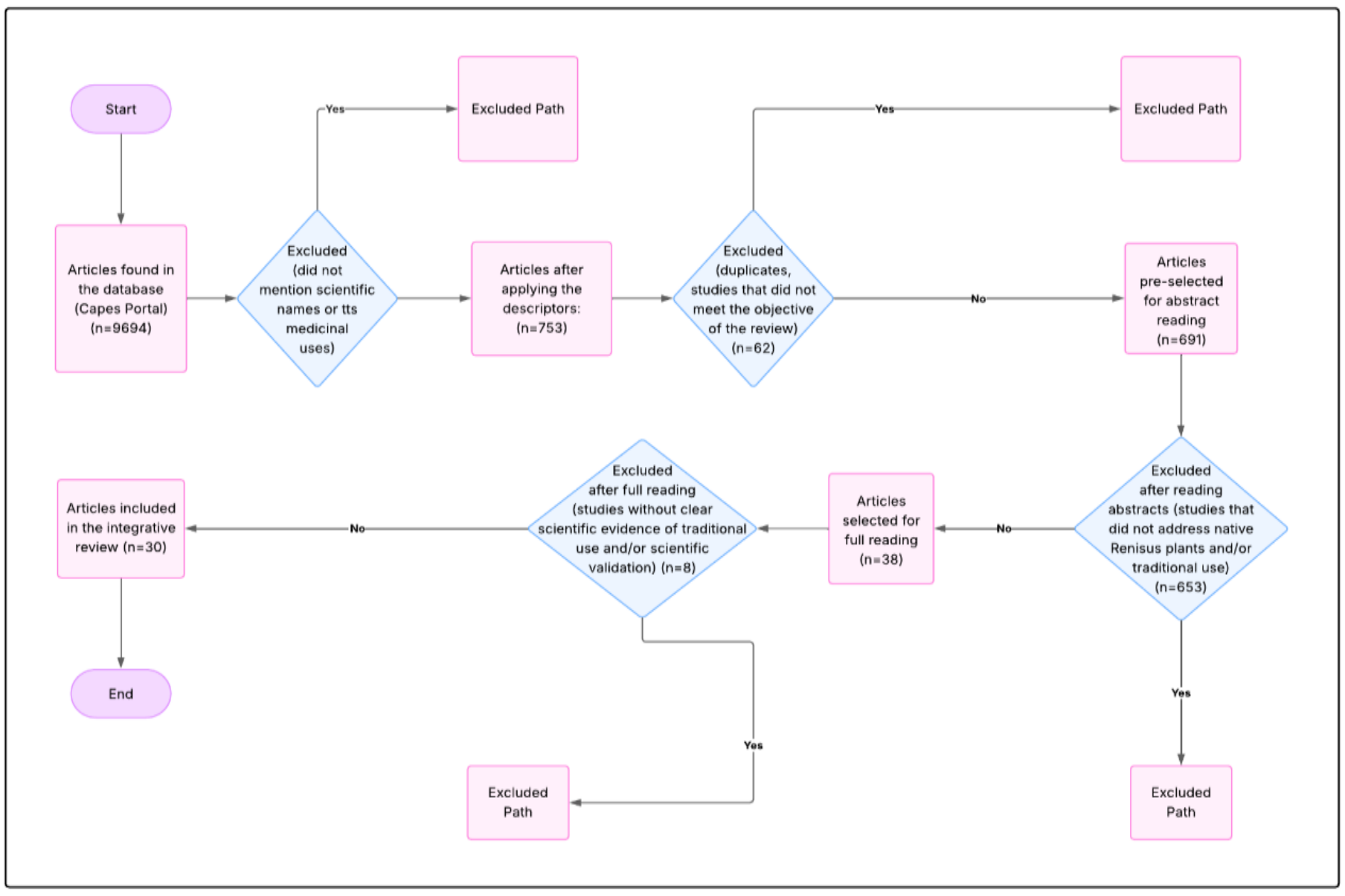

Figure 1. Left-to-right flowchart of study selection. Starts with 'Articles found (n=…)'. First decision sends off-topic records to an 'Excluded path'. Main line: 'After title/abstract screening (n=…)' → decision excluding non-relevant papers → 'After applying exclusion criteria (n=…)' → decision on evidence/validity → 'Selected for full-text (n=…)'. Final decision diverts ineligible full texts to exclusion; main line ends at 'Articles included in the integrative review (n=…)', then 'End'. 'Yes' continues; 'No' excludes.
