# Supplementary material for: Traditional medicinal plants in the Unified Health System of Brazil: a systematic review
Source: Front Pharmacol. 2026 Oct 21;17:1867902. doi: 10.3389/fphar.2026.1867902 (PMC13731864; doi:10.3389/fphar.2026.1867902)
Supplement: Supplementary file 2 [file Table1.docx]

# 1 Supplementary material

**Table 1.** Evidence Levels for RENISUS Plants.

| **Level of Scientific Evidence** | **Species** | **Predominant Type of Evidence** |
| --- | --- | --- |
| High (Includes human clinical studies) | Anacardium occidentale, Ananas comosus, Bidens pilosa, Copaifera sp., Tabebuia avellanedae | Clinical trials, case reports, intervention studies for inflammatory, metabolic, oncological, and wound conditions |
| Moderate (in vitro and in vivo studies) | Casearia sylvestris, Baccharis trimera, Lippia sidoides, Maytenus ilicifolia, Stryphnodendron adstringens, Eugenia uniflora, Justicia pectoralis | Preclinical evidence of antioxidant, anti-inflammatory, hepatoprotective, and antimicrobial activity |
| Low or Emerging (Limited or exploratory references) | Apuleia ferrea, Arrabidaea chica, Bauhinia spp., Carapa guianensis, Cordia spp., Costus spp., Croton spp., Dalbergia subcymosa, Eleutherine plicata, Erythrina mulungu, Mikania spp., Orbignya speciosa, Passiflora spp., Polygonum spp., Schinus terebinthifolius, Solanum paniculatum, Solidago microglossa, Uncaria tomentosa, Vernonia spp. | Sparse clinical data; mostly preliminary in vivo or ethnobotanical references |

**Table 2:** Studies in Humans on the Most Frequently Mentioned Medicinal Plants

| **Plant** | **Study** | **Reference** |
| --- | --- | --- |
| Ananas comosus | Evaluate the use of bee honey associated with Bromelin to treat acute irritative cough. | (Peixoto et al., 2016) |
| Ananas comosus | Examine multimodal care for headaches, lumbopelvic pain and dysmenorrhea in a patient with endometriosis. | (Martin, 2021) |
| Ananas comosus | Evaluate the effect of pineapple juice on postpartum uterine involution. | (Batubara & Pulungan, 2022) |
| Ananas comosus | Investigate the pharmacological properties of Ananas comosus on uterine activity. | (Monji et al., 2016) |
| Ananas comosus | Evaluate the effect of Ananas comosus on perineal pain and healing after episiotomy. | (Golezar, 2016) |
| Ananas comosus | Study the effect of a beverage containing several plants, including Ananas comosus, on lipid and glycemic profile. | (Dolores et al., 2018) |
| Ananas comosus | Investigate the penetration of bromelain into the blood and sinonasal mucosa in chronic rhinosinusitis. | (Passali et al., 2018) |
| Bidens pilosa | Evaluate the effectiveness of a formulation containing curcuminoids and Bidens pilosa for oral mucositis induced by cancer treatment. | (Arantes et al., 2021) |
| Bidens pilosa | Examine the effects of a Bidens pilosa formulation on blood homeostasis and β-cell function in men. | (Lai et al., 2015) |
| Bidens pilosa | Test a mucoadhesive formulation of curcuminoids and Bidens pilosa for the prevention and treatment of oral mucositis. | (Santos Filho et al., 2018) |
| Bidens pilosa | Evaluate the effects of a mucoadhesive phytomedicine on oral mucositis and quality of life in patients with head and neck cancer. | (Martins et al., 2023) |
| Copaifera sp. | Study the healing of chronic wounds with copaiba oleoresin. | (Arroyo-Acevedo et al., 2011) |
| Copaifera sp. | Report a case of hepatotoxicity associated with the use of Copaifera langsdorffii with Hypericum perforatum. | (Agollo et al., 2014) |
| Tabebuia avellanedae | Evaluate the safety and tolerability of Pau d’Arco in adults with primary dysmenorrhea. | (McClure et al., 2022) |
| Tabebuia avellanedae | Case report on the use of Tabebuia avellanedae in the treatment of esophageal cancer. | (Masaaki & Youichi, 2015) |
| Tabebuia avellanedae | Case report on the use of Tabebuia avellanedae in large B-cell lymphoma. | (Hideto, 2017) |
| Tabebuia avellanedae | First case report on the use of Tabebuia avellanedae in the treatment of metastatic thyroid cancer. | (Hideto, 2016) |

**Table 3**. Ananas comosus^2^

| Article Title | | Plant Part Used | | Sample | | Research Findings | |
| --- | --- | --- | --- | --- | --- | --- | --- |
| Use of honey associated with Ananas comosus (Bromelain) in the treatment of acute irritative cough (Peixoto et al., 2016). | Bromelain | | 50 | | The combination of honey and bromelain showed no significant difference in treating acute irritative cough compared to the control group. | |  |
| Multimodal Care for Headaches, Lumbopelvic Pain, and Dysmenorrhea in a Woman With Endometriosis: A Case Report (Martin, 2021) | | Not specified | | 1 | | The case report did not specifically focus on Ananas comosus but discussed multimodal treatment for endometriosis-related pain, including approaches that may involve bromelain or pineapple juice. | |
| The Effect of Pineapple Juice (Ananas comosus) on Uterine Involution in Postpartum Mothers at Pratama Jannah and Dermawati Maternity Clinics of Deli Serdang Regency in 2019 (Batubara & Pulungan, 2022). | | Fruit juice | | 100 | | Pineapple juice consumption accelerated uterine involution in postpartum mothers, leading to faster uterine size reduction and more efficient recovery compared to the control group. | |
| Investigation of Pharmacological Properties of Ananas comosus Extracts on Uterine Activity (Monii et al., 2016). | | Various extracts | | 30 | | Ananas comosus extracts demonstrated uterotonic effects, contributing to more efficient uterine activity, which may aid postpartum recovery. | |
| Ananas comosus Effect on Perineal Pain and Wound Healing After Episiotomy: A Randomized Double-Blind Placebo-Controlled Clinical Trial (Golezar, 2016). | | Bromelain (stem and fruit) | | 60 | | Bromelain accelerated perineal wound healing and significantly reduced post-episiotomy pain compared to the placebo group. | |
| Effect of a Beverage Containing Physalis peruviana, Passiflora edulis, Ananas comosus, Avena sativa, Linum usitatissimum, and Stevia rebaudiana on Lipid Profile and Glycemia in Overweight and Obese Women (Dolores et al., 2018). | | Plant mixture (including Ananas comosus) | | 80 | | The beverage, containing Ananas comosus, led to improvements in lipid profile and blood glucose in overweight and obese women, suggesting beneficial metabolic health effects. | |
| Bromelain's Penetration into the Blood and Sinonasal Mucosa in Patients with Chronic Rhinosinusitis (Passali et al., 2018). | | Bromelain (from Ananas comosus) | | 40 | | Bromelain effectively penetrated the sinonasal mucosa and bloodstream, providing relief from chronic rhinosinusitis symptoms, with significant effects compared to placebo. | |

**Table 4.** Bidens Pilosa^2^

| Study | Sample Size | Study Type | Findings |
| --- | --- | --- | --- |
| Safety and efficacy of a mucoadhesive phytomedication containing curcuminoids and Bidens pilosa in the prevention and treatment of oral mucositis (Arantes et al., 2021). | 120 | Triple-blind, randomized, placebo-controlled clinical trial | FITOPROT reduced OM severity from day 15 until the end of RT, while placebo showed increased severity (p < 0.05). The intervention group had fewer patients with ulcerative OM at the end of RT (p < 0.05). The phytomedication prevented increases in IL-8 levels and reduced salivary nitrite during RT. |
| Bidens pilosa Formulation Improves Blood Homeostasis and β-Cell Function in Men: A Pilot Study (Lai et al., 2015). | 14 | Pilot study | Improved blood glucose regulation and increased insulin secretion, suggesting benefits for glycemic control and β-cell function in men. |
| Effects of a Mucoadhesive Phytomedicine (Curcuma longa and Bidens pilosa) on Radiotherapy-Induced Oral Mucositis (Martins et al., 2023). | 52 | Randomized clinical trial | No statistical differences were found between groups for ROM assessment. Both groups showed worsened quality of life during RT. No statistically significant differences were observed between groups for any of the evaluated measures. |
| Randomized clinical trial of a mucoadhesive formulation containing curcuminoids (Zingiberaceae) and Bidens pilosa Linn (Asteraceae) extract (FITOPROT) for prevention and treatment of oral mucositis (Santos Filho et al., 2018). | 20 | Randomized clinical trial | Observed side effects included temporary, low-intensity mucosal/dental surface pigmentation (n = 7) and tooth sensitivity (n = 4), which resolved after discontinuation. No significant cellular genotoxic effects were observed (p > 0.05), and micronucleus frequencies remained unchanged (p > 0.05). |

^2^ The included studies employ diverse designs, such as triple-blind randomized controlled trials, randomized trials, and pilot studies, with varying doses, comparators, and endpoints; therefore, direct comparison of results is not feasible. FITOPROT is a multi-ingredient mucoadhesive formulation containing curcuminoids and Bidens pilosa, so observed effects cannot be attributed to Bidens pilosa alone. Abbreviations used include OM for oral mucositis and RT for radiotherapy. Reported adverse effects, such as transient pigmentation and tooth sensitivity, resolved following discontinuation of treatment. These summaries are intended to be descriptive and do not constitute clinical guidance.

**Table 5.** Copaifera sp.

| **Study** | **Part of the plant used** | **Number of participants** | **Results** |
| --- | --- | --- | --- |
| Healing of chronic wounds treated with copaiba oleoresin (Arroyo-Acevedo et al., 2011). | Resinous product extracted from the trunk of the copaiba tree | 6 | Significant acceleration of chronic wound healing, demonstrating the therapeutic potential of the oleoresin. |
| Hepatotoxicity induced by Hypericum perforatum with possible association with copaiba (Copaifera langsdorffii) (Agollo et al., 2014) | Herbal medicine | 1 | Case report of hepatotoxicity, suggesting a drug interaction between Hypericum perforatum and Copaifera langsdorffii, highlighting the importance of further safety studies. |

**Table 6.** Tabebuia avellanedae

| **Article Title** | **Plant Part Used** | **Number of Participants** | **Research Findings** |
| --- | --- | --- | --- |
| Safety and tolerability of Pau d'Arco (Tabebuia avellanedae) for primary dysmenorrhea: A single-arm, open-label trial on adults ages 18-45 (McLure et al., 2022). | Tabebuia avellanedae extract | 12 | The study showed that Pau d'Arco was safe and well-tolerated, with significant reduction in dysmenorrhea symptoms among participants. |
| Tabebuia avellanedae and Esophageal Cancer: A Case Report (Masaaki & Youichi, 2015). | Tabebuia avellanedae extract | 1 | Case report describing a positive response in an esophageal cancer patient using complementary treatment with Tabebuia avellanedae. |
| Tabebuia avellanedae and Diffuse Large B-cell Lymphoma: A Case Report (Hideto, 2017). | Tabebuia avellanedae extract | 1 | Case report suggesting Tabebuia avellanedae's potential as adjuvant therapy for B-cell lymphoma, with observed patient improvement. |
| Metastatic Thyroid Cancer Treated with Tabebuia avellanedae: A First Case Report (Hideto, 2016). | Tabebuia avellanedae extract | 1 | First reported case of Tabebuia avellanedae use in metastatic thyroid cancer, indicating positive disease control response. |

“*Tabebuia avellanedae* extract” (Pau d’arco) may come from different bark parts and solvents, with variable naphthoquinone content and doses. Oncology cases used the extract as complementary therapy

**Table 7**. Anacardium Occidentale

| Article Title | Plant Part Used | Sample Size | Research Findings |
| --- | --- | --- | --- |
| Effects of Cashew Nut (Anacardium occidentale L.) Seed Flour in Moderately Malnourished Children: Randomized Clinical Trial (Pereira de Jesus Costa et al., 2020). | Cashew nut seed flour | 40 | Cashew nut seed flour improved nutritional profile and aided in the recovery of moderately malnourished children, showing improvements in glycated hemoglobin and lipid profile. |
| Anacardium occidentale L. extract in cosmetic formulations: benefits for oily skin (Mercurio et al., 2017). | Anacardium occidentale extract | 50 | Anacardium occidentale extract demonstrated benefits for oily skin in cosmetic formulations, with positive effects on reducing oiliness and improving overall skin appearance. |
| Alpha-Amylase Inhibition and Antioxidative Capacity of Some Antidiabetic Plants Used by the Traditional Healers in Southeastern Nigeria (Oyedemi et al., 2017) | Traditional antidiabetic plants | Not specified | Some antidiabetic plants used in Nigeria showed alpha-amylase inhibition and antioxidant capacity, suggesting their beneficial properties in blood glucose control and diabetes prevention. |
| Phase II clinical pharmacological trial of a cream containing crude extract from Anacardium occidentale L. bark in the treatment of inflammatory wrist joint lesions (Padilha et al., 2020). | Crude extract from cashew nut bark | 30 | The cream containing crude extract from cashew nut bark was effective in treating inflammatory wrist joint lesions, with a significant reduction in inflammatory symptoms. |
| Nut-enriched energy-restricted diet has potential to decrease hunger in women at cardiometabolic risk: a randomized controlled trial (Brazilian Nuts Study) (Rocha et al., 2023). | Brazilian nuts (including Anacardium occidentale) | 100 | The calorie-restricted diet enriched with Brazilian nuts showed potential in reducing hunger in women at cardiometabolic risk, suggesting benefits in weight management and appetite control. |
